# Birth Weight, Childhood and Young Adult Overweight, and the Risk of Coronary Heart Disease in Men

**DOI:** 10.1161/ATVBAHA.123.320095

**Published:** 2023-11-16

**Authors:** Rebecka Bramsved, Maria Bygdell, Jari Martikainen, Staffan Mårild, Ingela Lindh, Annika Rosengren, Claes Ohlsson, Jenny M. Kindblom

**Affiliations:** Department of Pediatrics, Institute of Clinical Sciences (R.B., S.M.), The Sahlgrenska Academy, University of Gothenburg, Sweden.; Centre for Bone and Arthritis Research, Department of Internal Medicine and Clinical Nutrition, Institute of Medicine (R.B., M.B., C.O., J.M.K.), The Sahlgrenska Academy, University of Gothenburg, Sweden.; Bioinformatics and Data Centre (J.M.), The Sahlgrenska Academy, University of Gothenburg, Sweden.; Department of Obstetrics and Gynecology, Institute of Clinical Sciences (I.L.), The Sahlgrenska Academy, University of Gothenburg, Sweden.; Department of Molecular and Clinical Medicine, Sahlgrenska Academy at Gothenburg University, Sweden (A.R.).; Department of Drug Treatment, Sahlgrenska University Hospital, Region Västra Götaland, Gothenburg, Sweden (C.O., J.M.K.).

**Keywords:** birth weight, body mass index, coronary disease, myocardial ischemia, overweight, young adult

## Abstract

**BACKGROUND:**

Low birth weight is a known risk factor for adult coronary heart disease (CHD), but the additional effect of weight development during childhood and early adult life has not been studied.

**METHODS:**

We included 35 659 men born 1945 to 1961 from the population-based BMI Epidemiology Study Gothenburg, with data available on birthweight, BMI in childhood (8 years), and BMI in young adulthood (20 years). Information on CHD diagnoses was retrieved from national registers. We used Cox proportional hazards regression to estimate hazard ratios and 95% CIs for the risk of early and late CHD (before and after 58.4 years of age, respectively).

**RESULTS:**

During follow-up, a total of 3380 cases of CHD (fatal and nonfatal) were registered. Birth weight was inversely associated with the risk of both early (hazard ratio, 0.88 per SD increase [95% CI, 0.84–0.92]) and late (hazard ratio, 0.94 per SD increase [95% CI, 0.90–0.98]) CHD, independently of BMI at 8 years and BMI change during puberty. In a model including birth weight (below or above the median) together with overweight at 8 and 20 years, only birth weight and young adult overweight, but not overweight in childhood, were significantly associated with the risk of CHD. A birth weight below the median, followed by overweight at 20 years of age was associated with a more than doubled risk of early CHD (hazard ratio, 2.29 [95% CI, 1.86–2.81]), compared with the reference (birth weight above the median and normal weight at 20 years of age). This excess risk was even more pronounced for a birthweight below 2.5 kg.

**CONCLUSIONS:**

We demonstrate that low birth weight and young adult overweight are important developmental markers of risk for adult CHD. These findings motivate a life course perspective for prevention and risk assessment of adult CHD.

**Highlights**

- In a large population-based cohort of 35 659 men, birth weight was inversely related to the risk of coronary heart disease independent of body mass index at age 8 and age 20.
- In a model including birth weight, childhood, and young adult overweight, only birth weight and young adult overweight, but not childhood overweight, were significantly associated with the risk of coronary heart disease.
- A birth weight below the median (3.6 kg) followed by overweight at age 20 was associated with a more than doubled risk of adult coronary heart disease, compared with the reference group with birth weight above the median and young adult normal weight.

Coronary heart disease (CHD) is a leading cause of mortality and disease burden in all regions of the world.^1^ The prevalence of CHD is rising in low- and middle-income countries, and, worryingly, previously declining trends in some high-income countries may now be reversing.^1^ The obesity epidemic is believed to be a major contributing factor to the increase in CHD prevalence.^2^ In 2015, it was estimated that high BMI accounted for 4.0 million deaths, more than two thirds of which were caused by cardiovascular disease.^3^ Given the long latency periods for the disease processes behind established CHD, early life risk factors and risk accumulation across the life course are likely to be of importance.

The Developmental Origins of Health and Disease hypothesis states that exposure to environmental stress during a critical period of development can increase the risk of disease decades later in life. David Barker, the leading proponent of the theory, has described that suboptimal intrauterine conditions leading to growth restriction and manifested as low birth weight, were associated with higher risk of CHD in adulthood.^4^ High BMI in childhood and adolescence has also been reported to be associated with a higher risk of CHD events.^5^ However, the relative contribution of low birth weight together with elevated BMI at successive stages of development for the risk of CHD has not been addressed.

The population-based BEST (BMI Epidemiology Study) cohort in Gothenburg, Sweden,^6^ with birth weight and BMI during development collected from school health records and the conscription examination, and with information on diagnoses and causes of death available through linkage with high-quality national registers, offers a unique possibility to address these questions. The aim of the present study was to evaluate the risk of CHD in relation to birth weight and BMI during childhood and puberty as well as during early adult life in a cohort of Swedish men born 1945 to 1961.

## METHODS

In accordance with the Transparency and Openness Promotion Guidelines, anonymized data that are minimally required to reproduce results can be made available from the corresponding author upon reasonable request, and upon approval from the University of Gothenburg according to mandatory national law.

### Study Population and Data Collection

The population-based BEST Gothenburg cohort was initiated with the overall aim to study the impact of BMI during childhood and puberty on adult diseases, as previously described.^6^ In the present study, we collected information on birth weight together with developmental weight and height from regular visits to child health care centers and school health care for all men born 1945 to 1961 in Gothenburg, Sweden. During this period, school was mandatory from 7 years of age and the attendance to school health care was >98.5%.^7^ We also retrieved weight and height at young adult age from military conscription tests, mandatory until 2010 for all Swedish men, for individuals in the cohort.^8^ Eligible individuals were men with a 10-digit personal identity number and a school health record in the central archive. Figure S1 shows the inclusion of individuals in the present study. Men with data available on birth weight, childhood BMI, and young adult BMI were included (n=35 659) and followed from the age of 20 years until censoring due to being registered with a CHD diagnosis, death, migration, or until December 31, 2019, whichever occurred first. Individuals with a first CHD diagnosis, or who died or emigrated before the age of 20 were not included in this study. The Ethics Committee of the University of Gothenburg, Sweden approved the study and waived the need for written consent.

### Exposures

Childhood BMI and young adult BMI were calculated using all paired weight and height measurements between 6.5 and 9.5 years of age for childhood BMI, and between 17.5 and 22.0 years of age for young adult BMI. Both BMI variables were age-adjusted for each individual participant using a linear regression model with BMI as the dependent variable and age as an independent variable. The age-dependent change was assumed to follow the slope of the fitted models, and BMI at exactly 8 years for childhood BMI and 20 years for young adult BMI was estimated using the slope of the fitted models.^6^ Pubertal BMI change was defined as the difference between young adult BMI and childhood BMI (BMI at age 20 − BMI at age 8).

Childhood overweight (including obesity) was defined according to the cutoff limits for sex and age at 8 years defined by Centers for Disease Control and Prevention^9^ and for young adult age as BMI ≥25 kg/m^2^.

### Linkage With Registers and Study Outcomes

Dates and diagnoses of CHD were retrieved by linking the cohort with registers held by the National Board of Health and Welfare: The National Patient Register and the Cause of Death Register. The National Patient Register started in 1964 with full coverage of the Gothenburg region from 1972. The Cause of Death Register contains information on causes of death since 1961 and covers the entire follow-up period of this study. CHD diagnoses were classified according to the *International Classification of Diseases system (ICD*), diagnostic codes I20-I25 in ICD10, 410 to 414 in ICD9, and 410 to 413 in ICD8. We used inpatient or outpatient visits where CHD was recorded as the main diagnosis for the first time.

Using the unique 10-digit personal identity number assigned to every Swedish citizen at birth or immigration, the data in the BEST Gothenburg cohort were linked to the Longitudinal Integration Database for Health Insurance and Labor Market Studies at Statistics Sweden. The country of birth of the study subjects and their parents were retrieved, as well as the subjects’ highest education level until 45 years of age. Education level was defined as low (elementary school), middle (secondary school), or high (postsecondary education).

### Statistical Analyses

There were no missing values for the main variables in the included cohort (birth weight, childhood BMI, young adult BMI, birth year, country of birth, or outcome). Descriptive data are presented as means, SD, medians, interquartile range, and range. Categorical variables are presented as numbers and percentages.

We used Cox proportional hazards regression, with follow-up starting at age 20 years, to estimate hazard ratios (HRs) and 95% CIs for the association between exposures and events, with all analyses adjusted for birth year and country of birth unless otherwise stated. The assumption of proportional hazards was evaluated by visual inspection of Schoenfeld residual plots and through proportional hazards test. Possible interactions were evaluated by addition of an interaction term (the variables of interest multiplied with each other) in the Cox regression models (*P*<0.05 for an interaction term was interpreted as a statistically significant interaction). A Kaplan-Meier survival plot was performed with study participants divided into 4 groups by median birth weight and young adult overweight/normal weight and inference was evaluated using the log-rank test between the 4 groups. In sensitivity analyses, we repeated the analyses using logistic regression. Moreover, we tested the associations after truncation of the cohort to include only individuals with birth weights within the normal range (2.50–4.50 kg).^10,11^ We also performed analyses with additional adjustment for education level.

Kaplan-Meier plots and proportional hazards test were performed in R (Version 4.3.0) using the survival package v3.5-5.^12^ For all other statistical analyses, SPSS Version 28.0.1.0 was used.

## RESULTS

### Study Population and Incidence of CHD

After exclusions, this population-based study included 35 659 men born between 1945 and 1961 with information available on birth weight, childhood BMI at 8 years, and young adult BMI at age 20, followed until December 31, 2019 (Figure S1). The mean follow-up from age 20 was 41.8 years (SD, 10.3 years), with a total of 1 490 500 person-years of follow-up. There were 3380 cases of CHD (fatal and nonfatal) before the end of follow-up. The median age at first CHD diagnosis was 58.4 years (Table 1).

**Table 1.**
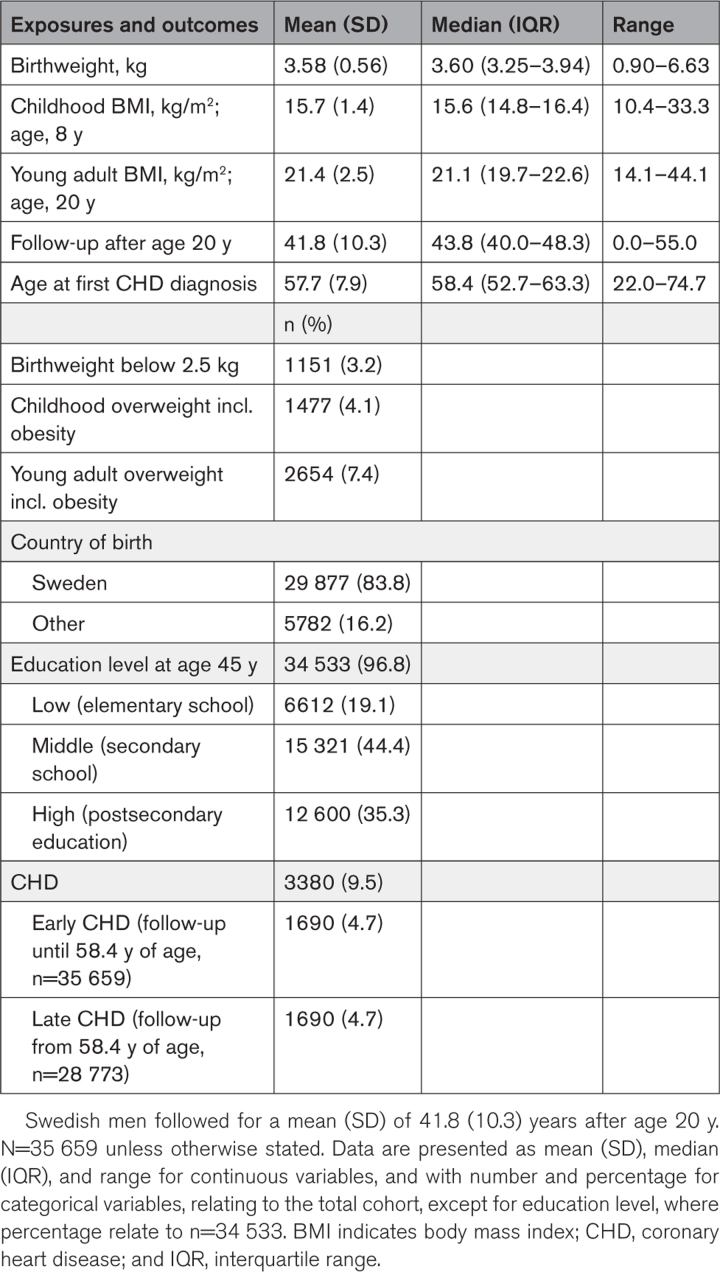
Cohort Description

In a correlation analysis, birthweight showed a weak correlation with childhood BMI (Pearson *r*=0.18) and young adult BMI (Pearson *r*=0.09), indicating that birthweight only explains 3.3% of the variance in childhood BMI and 0.8% of the variance in young adult BMI.

### Associations Between Birth Weight and Developmental BMI and the Risk of CHD

Since we found indications of violation of the assumption of proportional hazards for the association between exposure and outcome, we stratified the follow-up on the median age at diagnosis (age, 58.4) to evaluate associations with early and late CHD. In a Cox proportional hazards regression model adjusted for birth year and country of birth, birth weight was inversely associated with the risk of both early and late CHD. This association was maintained when BMI at 8 years and the pubertal BMI change were included in the model (Table 2). The increase in risk in relation to the developmental parameters was more pronounced for early than late CHD for all examined exposures.

**Table 2.**
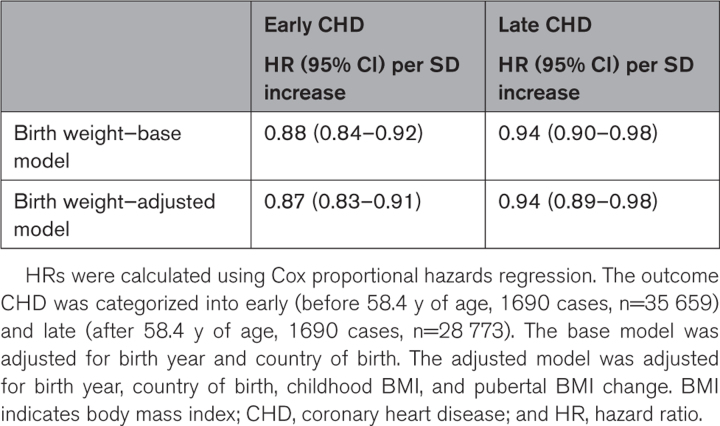
Adjusted HRs for CHD in Relation to Birth Weight, Childhood BMI, and Pubertal BMI Change in 35 659 Swedish Men Followed for a Mean of 41.8 (10.3) Years After Age 20 Years

### Risk of CHD in Relation to Birth Weight and Developmental Overweight

To further evaluate the importance of birth weight in combination with developmental BMI status, we dichotomized the study population based on the birth weight median and overweight status at 8 (childhood) and 20 years (young adult age). A birth weight below the median (<3.6 kg) was associated with higher risk of both early and late CHD compared with a birth weight above the median (≥3.6 kg; Table 3). In a model including birth weight together with overweight at 8 and 20 years, only birth weight and young adult overweight were significantly associated with early and late CHD (Table 3). These findings establish low birth weight and young adult overweight as developmental markers of the risk for CHD events, and we therefore included only these 2 risk markers in the subsequent analyses.

**Table 3.**
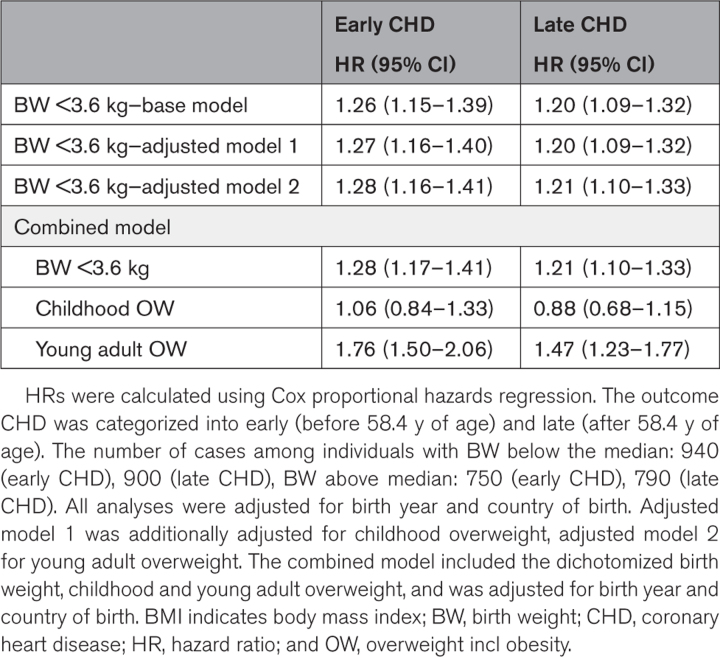
Adjusted HRs for CHD in Relation to Birth Weight, Childhood Overweight, and Young Adult Overweight Among 35 659 Swedish Men Followed for a Mean of 41.8 (10.3) Years After Age 20 Years

### Low Birth Weight and Young Adult Overweight as Risk Markers of Adult CHD

We next sought to evaluate the risk associated with combinations of birth weight and overweight status in young adulthood (Figure; Table 4). The group with a birth weight above the median and normal weight in young adult age was used as reference. A birth weight below the median followed by normal weight at young adult age, and a birth weight above the median followed by young adult overweight, were associated with increased risk of early and late CHD compared with the reference group (Table 4). A birth weight below the median followed by young adult overweight was associated with a pronounced excess risk of both early and late CHD, compared with the reference group (HR, 2.29 [95% CI, 1.86–2.81] for early and HR, 1.60 [95% CI, 1.24–2.08] for late).

**Table 4.**
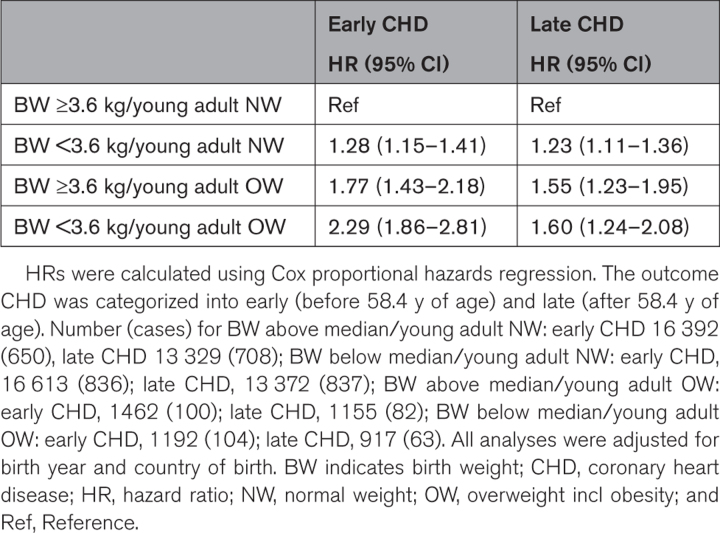
Adjusted HRs for CHD in Relation to Birth Weight and Young Adult Overweight Among 35 659 Swedish Men Followed for a Mean of 41.8 (10.3) Years After Age 20 Years

**Figure.**
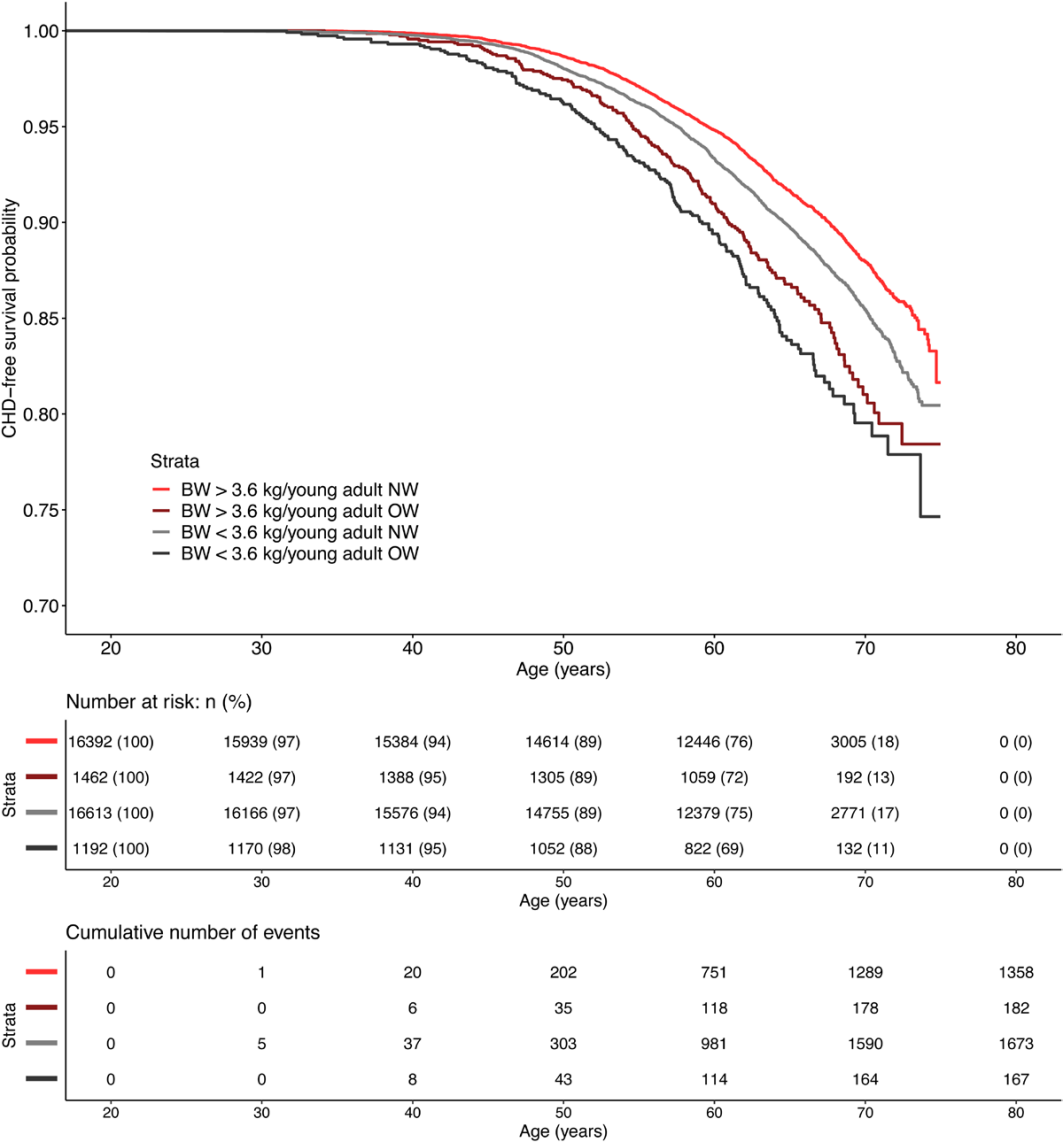
**Kaplan-Meier curve of survival free from coronary heart disease (CHD) in relation to birth weight above or below 3.6 kg, followed by normal weight or overweight (incl obesity) at 20 years of age in 35 659 men followed for a mean of 41.8 years.** The *P* value for comparison between all groups and the reference group (birthweight [BW] ≥3.6 kg followed by young adult normal weight [NW]) was <0.001. OW indicates overweight incl. obesity.

In a subanalysis including only men with a birth weight below 3.6 kg (n=17 805), young adult overweight was associated with a significantly increased risk of both early and late CHD (HR, 1.79 [95% CI, 1.46–2.20] for early CHD, HR, 1.30 [95% CI, 1.00–1.67] for late CHD).

In a Kaplan-Meier curve, all 3 groups differed significantly from the reference group. We observed that the group with a birth weight below 3.6 kg followed by young adult overweight was affected by CHD events already after 40 years of age, approximately 10 years earlier than the reference group (Figure).

### Additional Adjustments and Sensitivity Analyses

All additional analyses are shown in the Supplemental Material. To further examine the risk for adult CHD associated with low birth weight, we used the clinical cutoff for low birth weight, 2.5 kg,^10^ in combination with overweight at 20 years of age. The group with birth weight above 2.5 kg and young adult normal weight was used as reference group. Individuals with the combination of low birth weight followed by young adult overweight had a clear excess risk of early CHD (HR, 3.07 [95% CI, 1.70–5.56]), compared with the reference group (Table S1). For men with a birth weight below 2.5 kg (n=1151), adult overweight was associated with an increased risk for early but not late CHD (early: HR, 2.70 [95% CI, 1.41–5.15], late: HR, 0.88 [95% CI, 0.27–2.72]).

As a sensitivity analysis, we evaluated the association between birth weight and young adult BMI for the entire study period without considering the time to event, using a logistic regression model with CHD as outcome. When birth weight was included in a logistic regression model together with childhood BMI and BMI change during puberty, significant associations were seen for birth weight and BMI change during puberty, but not childhood BMI, with CHD (Table S2). In a model using the groups based on birth weight dichotomized on the median and young adult overweight, we found that both a birth weight below the median and young adult overweight were associated with the risk of CHD, compared with the reference group (the group with birth weight above the median and young adult normal weight). However, the most pronounced risk was seen for the combination of a birth weight below the median and young adult overweight (Table S2). These findings were in line with the results from the Cox regression models.

Results from analyses with birth weight restricted to the normal range of 2.5 to 4.5 kg (Tables S3 through S5) and with adjustment for education (Tables S6 through S8) were essentially similar to the main results.

## DISCUSSION

The prevalence of CHD is rapidly rising in low- and middle-income countries, and a recent rise has been observed in some high-income countries where age-standardized rates have previously been declining.^1^ Hence, there is a need to increase our understanding of which risk factors and changes in risk factor pattern might explain these developments. In the present study, we demonstrate that birth weight is inversely associated with early and late CHD, and this association is maintained also after adjustment for the postnatal BMI development using BMI during childhood and the pubertal BMI change. Importantly, a birth weight below the median, in combination with overweight as a young adult, was associated with a pronounced risk of early CHD, and an even higher excess risk was seen for individuals with a low birth weight (below 2.5 kg) followed by young adult overweight. In contrast, childhood overweight, when included in a model together with birth weight and young adult overweight, was not significantly associated with the risk of early or late CHD.

Previous observational evidence has shown associations between low birth weight and later cardiovascular disease in adulthood,^4,13,14^ mostly without considering BMI in childhood, puberty, or at the end of the developmental period; the young adult age. In the present study, we confirm the inverse association between birth weight and the risk of CHD and add to available evidence by demonstrating maintained associations between birth weight and adult CHD after accounting for BMI in childhood and adolescence. A few previous studies considered the association between birth weight together with young adult BMI for the risk of CHD. In the Nurses’ health study, the increased risk of CHD associated with high adult BMI was the highest among women with a self-reported birth weight below 2.5 kg.^15^ In a Danish Study, men with a birth weight below 2.5 kg and overweight at age 19 had >5× increased risk of CHD, compared with men with a birth weight between 2.5 and 3.9 kg and a BMI below 25 kg/m^2^ at 19 years.^16^ A small study in men using self-reported birth weight concluded that the increased risk of CHD associated with low birth weight was restricted to men who had high BMI in adulthood.^17^ In addition to these studies evaluating birth weight together with adult overweight, a Finnish study investigated the impact of low birth weight considering the BMI in childhood. The study demonstrated that the highest CHD death rates were seen for boys who were thin at birth but later caught up and were of average or above average BMI from seven years of age.^18^ Thus, there is evidence to support increased CHD risk for individuals with a birth weight below 2.5 kg in combination with either overweight in adult age, or with rapid catch-up growth resulting in normal or above normal BMI in childhood. However, no previous study has considered birth weight together with the entire developmental period (childhood and puberty, until young adult age). In the present study, we demonstrate that when birth weight is included in a Cox regression model together with overweight in childhood and young adulthood, only low birth weight and young adult overweight were significantly associated with the risk of CHD. These findings establish low birth weight and young adult overweight as important developmental markers of the risk for CHD and indicate that developing overweight during puberty is particularly detrimental to the risk of developing CHD. Our findings demonstrate an excess risk of developing early CHD among men with low birth weight followed by overweight in young adult age, and that this group is affected by CHD already from 40 years of age, approximately 10 years earlier than the reference group. In addition, we demonstrate that the inverse association between birth weight and the risk of CHD is present also within the birth weight interval of 2.5 to 4.5 kg, indicating that this association is not entirely driven by extremely low birth weights.

Of the examined exposures, childhood BMI displayed the weakest association with risk of CHD in the present study. In the combined model including childhood overweight together with birth weight and young adult overweight, childhood overweight was not significantly associated with the risk of CHD. Childhood BMI between 7 and 13 years of age has previously been reported to be associated with increased risk of CHD.^5^ When put in the context of birth weight and later BMI development, however, overweight in childhood is no longer significant. This is in line with previous studies also indicating that a large pubertal BMI change is of greater importance than childhood BMI for the risk of cardiovascular disease.^6,19^

A recent systematic review and meta-analysis suggested a J-shaped relationship between birth weight and cardiovascular risk.^20^ However, a study from the UK biobank could not confirm an increased risk of cardiovascular disease for high (self-reported) birth weight when middle-aged BMI was considered in a cohort of more than 250 000 individuals.^21^ A Mendelian randomization approach using the UK biobank cohort demonstrated a causal relationship between low birth weight and later CHD, not mediated via adult obesity.^22^ Our study, using an observational approach, demonstrated a clear excess risk for early CHD for individuals with low birth weight, especially in combination with weight gain during puberty resulting in young adult overweight.

Several mechanisms may explain the association between low birth weight in combination with young adult overweight with increased risk of CHD. Various adverse intrauterine conditions such as maternal smoking, hypertension, and poor nutritional conditions can result in a low birth weight.^23^ Poor nutrition in utero was suggested by Barker and Hales to induce the so-called thrifty phenotype as a compensatory response, leading to permanent changes in the glucose-insulin metabolism.^24^ Periconceptional starvation has been shown to cause epigenetic alterations supporting the hypothesis that low birth weight, as a proxy for poor intrauterine nutrition, entails lifelong metabolic consequences.^25^ Adult overweight is known to be associated with traditional major risk factors for CHD; hypertension, dyslipidemia, type 2 diabetes, but is also in itself an independent risk factor, through low-grade inflammation accelerating the atherosclerotic process of the intima media.^26^ Low birth weight has been observed to be associated with elevated blood pressure, arterial narrowing, and endothelial dysfunction already in childhood,^27,28^ and hence, it is likely that individuals with low birth weight are more sensitive to the adverse effects of adult obesity. Moreover, weight gain during adolescence, in contrast to excess weight present already in childhood, is characterized by an accumulation of visceral adipose tissue,^29,30^ known to be more metabolically deleterious than subcutaneous fat.^31^ Young adult weight has been shown to be associated with a higher prevalence of coronary atherosclerosis in middle-aged men and women in Sweden.^32^ One may speculate that our findings could, at least partly, be explained by earlier onset of the atherosclerotic process leading to earlier onset of CHD in the group with the combination of low birth weight and young adult overweight.

Our study presents a range of strengths including the well-powered population-based cohort with data on birth weight together with BMI during childhood and puberty, a low death rate before the age of 20 years, and with reliable register-based data on CHD outcomes with virtually no loss to follow-up. However, our study is limited by the lack of data for women, who generally have a later onset of CHD, and who might be less sensitive to a combination of low birth weight and adult obesity. We had the advantage of highly reliable register data on birth weight, but information on gestational age and maternal as well as own smoking was not available. Furthermore, a vast majority of the men in the study were of European descent. The results may therefore not be generalizable to other ethnicities.

In conclusion, the present study establishes that low birth weight and young adult overweight are important developmental markers of risk for CHD, a pattern that might be common in many low- and middle-income countries with a rapid increase in CHD. Considering that individuals with a birth weight below median who developed overweight in young adulthood had a doubled risk of developing early CHD, a life course perspective should be used for CHD prevention and risk assessment in adults.

## ARTICLE INFORMATION

### Sources of Funding

This study was supported by the Swedish Research Council (J.M. Kindblom: 2021-01439; C. Ohlsson: 2016-01001; A. Rosengren: 2018-02527), the Heart-Lung Foundation (J.M. Kindblom: 20220406; 20220620) and by grants from the Swedish state under the agreement between the Swedish government and the county councils, the ALF-agreement (J.M. Kindblom: ALFGBG-965996, C. Ohlsson: ALFGBG-720331), the Lundberg Foundation (C. Ohlsson: 2017-0081), the Torsten Söderberg Foundation (C. Ohlsson: M65/15), the Novo Nordisk Foundation (C. Ohlsson: NNF180C0033898), the Knut and Alice Wallenberg Foundation (C. Ohlsson: KAW, 2015.0317), the Swedish Society for Medical Research (M. Bygdell PD20-0012) Region Västra Götaland, Research and Development Primary Health Care (R. Bramsved: VGFOUSA-983036).

### Disclosures

I. Lindh has received compensation from Gedeon Richter and Exeltis for lectures and participation in Advisory Board during the previous 3 years. The other authors report no conflicts.

### Supplemental Material

Figure S1

Tables S1–S8

## Supplementary Material


