# Supplementary material for: Birth Weight, Childhood and Young Adult Overweight, and the Risk of Coronary Heart Disease in Men
Source: Arterioscler Thromb Vasc Biol. 2023 Nov 16;44(1):314–21. doi: 10.1161/ATVBAHA.123.320095 (PMC10749680; doi:10.1161/ATVBAHA.123.320095)
Supplement: Supplementary file 1 [file atv-44-314-s001.pdf]

Supplementary Material

Birth weight, childhood and young adult overweight and the risk of coronary heart disease in men

Rebecka Bramsved[1,2*], Maria Bygdell[2], Jari Martikainen[3], Staffan Mårild[1], Ingela Lindh[4], Annika Rosengren[5], Claes Ohlsson[2,6], Jenny M Kindblom[2,6]

Affiliations

[1]Department of Pediatrics, Institute of Clinical Sciences, The Sahlgrenska Academy, University of Gothenburg, Gothenburg, Sweden

[2]Centre for Bone and Arthritis Research, Department of Internal Medicine and Clinical Nutrition, Institute of Medicine, The Sahlgrenska Academy, University of Gothenburg, Gothenburg, Sweden

[3]Bioinformatics and Data Centre, The Sahlgrenska Academy, University of Gothenburg, Gothenburg, Sweden

[4]Department of Obstetrics and Gynecology, Institute of Clinical Sciences, The Sahlgrenska Academy, University of Gothenburg, Gothenburg, Sweden

[5]Department of Molecular and Clinical Medicine, Sahlgrenska Academy at Gothenburg University, Gothenburg, Sweden

[6]Department of Drug Treatment, Region Västra Götaland, Sahlgrenska University Hospital, Gothenburg, Sweden

*Corresponding author
Rebecka Bramsved
Department of Pediatrics
Queen Silvia Children's Hospital
416 85 Gothenburg, Sweden
Phone: +46707328915
E-mail: rebecka.bramsved@gu.se
Fax: +4632283785

DOI [to be added]

Figure S1. Flow chart of included individuals

PIN, personal identity number; BMI, body mass index

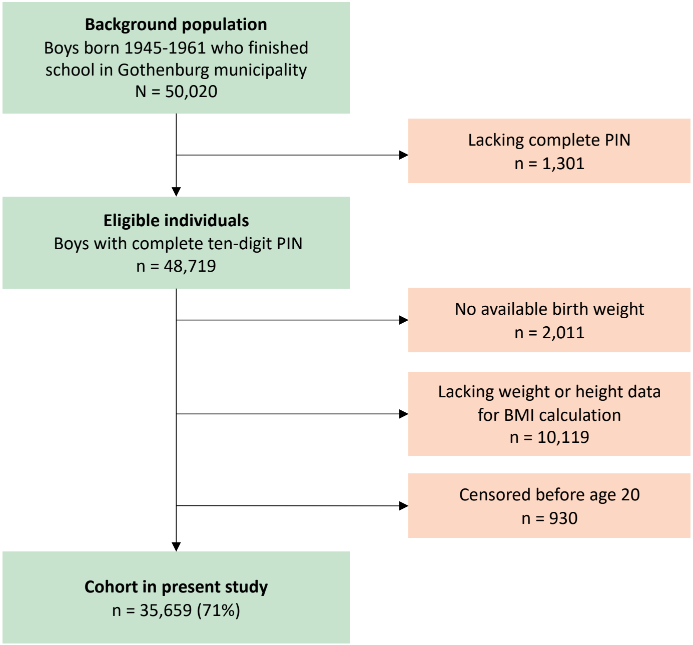

DOI [to be added]

Table S1. Adjusted Hazard Ratios for coronary heart disease (CHD) in relation to birth weight and young adult overweight in combined analyses among 35,659 Swedish men followed for a mean of 41.8 (10.3) years after age 20 years

	Early CHD HR (95% CI)	***Late CHD*** HR (95% CI)
BW ≥ 2.5 kg/young adult NW	ref	ref
BW < 2.5 kg/young adult NW	1.22 (0.94; 1.58)	1.19 (0.91; 1.56)
BW ≥ 2.5 kg/young adult OW	1.72 (1.48; 2.00)	1.42 (1.20; 1.70)
BW < 2.5 kg/young adult OW	3.07 (1.70; 5.56)	1.00 (0.32; 3.12)

Hazard Ratios (HRs) were calculated using Cox proportional hazards regression. The outcome CHD was categorized into early (before 58.4 years of age) and late (after 58.4 years of age). BW < 2.5 kg (n =1,151) for the full cohort. Number (cases) for BW ≥ 2.5 kg /young adult NW: Early CHD 31,935 (1,427), Late CHD 25,852 (1,489); BW < 2.5 kg /young adult NW: Early CHD 1,070 (59), Late CHD 849 (56); BW ≥ 2.5 kg / young adult OW: Early CHD 2,573 (193), Late CHD 2,015 (142); BW < 2.5 kg /young adult OW: Early CHD 81 (11), Late CHD 57 (3). All analyses were adjusted for birth year and country of birth.

CHD, coronary heart disease; BW, birth weight; NW, normal weight; OW, overweight incl obesity; CI, confidence interval.

DOI [to be added]

Table S2. Adjusted Odds Ratios for coronary heart disease (CHD) in relation to birth weight and young adult overweight among 35,659 Swedish men followed for a mean of 41.8 (10.3) years after age 20 years

	CHD OR (95% CI)
Birth weight	0.91 (0.88; 0.94)
Childhood BMI	1.03 (0.99; 1.07)
Pubertal BMI change	1.18 (1.14; 1.22)
BW ≥ 3.6 kg/young adult NW	ref
BW < 3.6 kg/young adult NW	1.27 (1.18; 1.37)
BW ≥ 3.6 kg/young adult OW	1.71 (1.45; 2.02)
BW < 3.6 kg/young adult OW	2.04 (1.71; 2.43)

Odds Ratios (ORs) were calculated using logistic regression. All analyses were adjusted for birth year and country of birth. Total number (cases of CHD): 35,659 (3,380)

CHD, coronary heart disease; BW, birth weight; NW, normal weight; OW, overweight incl obesity; CI, confidence interval.

DOI [to be added]

Table S3. Adjusted Hazard Ratios for coronary heart disease (CHD) in relation to birth weight, childhood BMI and pubertal BMI change, restricted to birth weights between 2.5 and 4.5 kg

	Early CHD HR (95% CI) per SD increase	***Late CHD*** HR (95% CI) per SD increase
Separate models		
Birth weight	0.86 (0.81; 0.92)	0.90 (0.84; 0.96)
Childhood BMI	1.07 (1.02; 1.13)	1.01 (0.96; 1.06)
Pubertal BMI change	1.25 (1.20; 1.31)	1.11 (1.06; 1.17)
Combined model		
Birth weight	0.85 (0.80; 0.91)	0.90 (0.84; 0.96)
Childhood BMI	1.07 (1.02; 1.12)	1.02 (0.96; 1.07)
Pubertal BMI change	1.24 (1.19; 1.30)	1.11 (1.05; 1.17)

Hazard Ratios (HRs) were calculated using Cox proportional hazards regression. N = 32,676

The outcome CHD was categorized into early (before 58.4 years of age, 1,553 cases, n = 32,676) and late (after 58.4 years of age, 1,553 cases, n = 25,354). All analyses were adjusted for birth year and country of birth with either birth weight, childhood BMI or pubertal BMI included in the separate models, and birth weight, childhood BMI and pubertal BMI included together in the combined model.

CHD, coronary heart disease; BMI, body mass index, CI, confidence interval, SD, standard deviation.

DOI [to be added]

Table S4. Adjusted Hazard Ratios for coronary heart disease (CHD) in relation to birth weight and young adult overweight, restricted to birth weights between 2.5 and 4.5 kg

	Early CHD HR (95% CI)	***Late CHD*** HR (95% CI)
BW < 3.6 kg **–** *base model*	1.22 (1.10; 1.35)	1.21 (1.09; 1.33)
BW < 3.6 kg - *adjusted model 1*	1.23 (1.11; 1.36)	1.21 (1.09; 1.33)
BW < 3.6 kg - *adjusted model 2*	1.23 (1.11; 1.36)	1.21 (1.10; 1.34)
Combined model		
BW < 3.6 kg	1.23 (1.12; 1.37)	1.21 (1.09; 1.34)
Childhood OW	1.06 (0.83; 1.34)	0.90 (0.68; 1.18)
Young Adult OW	1.77 (1.50; 2.08)	1.47 (1.22; 1.78)

Hazard Ratios (HRs) were calculated using Cox proportional hazards regression. N = 32,676. The outcome CHD was categorized into early (before 58.4 years of age) and late (after 58.4 years of age). The number of cases among individuals with BW below the median: 885 (early CHD), 837 (late CHD), BW above median: 668 (early CHD), 703 (late CHD). All analyses were adjusted for birth year and country of birth. Adjusted model 1 was additionally adjusted for childhood overweight, adjusted model 2 for young adult overweight. The combined model included the dichotomized birth weight, childhood and young adult overweight and was adjusted for birth year and country of birth.

CHD, coronary heart disease; BW, birth weight; OW, overweight incl obesity; CI, confidence interval.

DOI [to be added]

Table S5. Adjusted Hazard Ratios for coronary heart disease (CHD) in relation to birth weight and young adult overweight restricted to birth weights between 2.5 and 4.5 kg

	Early CHD<br>HR (95% CI)	***Late CHD***<br>HR (95% CI)
BW ≥ 3.6 kg/young adult NW	ref	ref
BW < 3.6 kg/young adult NW	1.24 (1.11; 1.38)	1.23 (1.10; 1.36)
BW ≥ 3.6 kg/young adult OW	1.83 (1.47; 2.28)	1.53 (1.20; 1.96)
BW < 3.6 kg/young adult OW	2.16 (1.74; 2.70)	1.65 (1.26; 2.15)

Hazard Ratios (HRs) were calculated using Cox proportional hazards regression. N = 32,676 The outcome CHD was categorized into early (before 58.4 years of age) and late (after 58.4 years of age). Number (cases) for BW above median/young adult NW: Early CHD 14,840 (599), Late CHD 12,046 (633); BW below median/young adult NW: Early CHD 15,437(768), Late CHD 12,436 (777); BW above median/ young adult OW: Early CHD 1,293 (93), Late CHD 1,015 (70); BW below median/young adult OW: Early CHD 1,106 (93), Late CHD 857 (60). All analyses were adjusted for birth year and country of birth.

CHD, coronary heart disease; BW, birth weight; NW, normal weight; OW, overweight incl obesity; CI, confidence interval.

DOI [to be added]

Table S6. Adjusted Hazard Ratios for coronary heart disease (CHD) in relation to birth weight, childhood BMI and pubertal BMI change, adjusted for education level

	Early CHD HR (95% CI) per SD increase	***Late CHD*** HR (95% CI) per SD increase
Separate models		
Birth weight	0.89 (0.85; 0.93)	0.94 (0.90; 0.99)
Childhood BMI	1.07 (1.02; 1.12)	1.01 (0.96; 1.06)
Pubertal BMI change	1.23 (1.18; 1.28)	1.08 (1.03; 1.14)
Combined model		
Birth weight	0.88 (0.84; 0.92)	0.94 (0.90; 0.99)
Childhood BMI	1.07 (1.02; 1.12)	1.02 (0.97; 1.07)
Pubertal BMI change	1.22 (1.17; 1.27)	1.08 (1.03; 1.13)

Hazard Ratios (HRs) were calculated using Cox proportional hazards regression. N = 34,533.

The outcome CHD was categorized into early (before 58.4 years, n = 34,533, 1,681 cases) and late (after 58.4 years, n = 28,696, 1,683 cases). All analyses were adjusted for birth year, country of birth and education level at 45 years of age, with either birth weight, childhood BMI or pubertal BMI change included in the separate models, or with birth weight, childhood BMI and pubertal BMI change included together in the combined model.

CHD, coronary heart disease; BMI, body mass index, CI, confidence interval, SD, standard deviation.

DOI [to be added]

Table S7. Adjusted Hazard Ratios for coronary heart disease (CHD) in relation to birth weight and young adult overweight, adjusted for education level

	Early CHD HR (95% CI)	***Late CHD*** HR (95% CI)
BW < 3.6 kg **–** *base model*	1.26 (1.14; 1.38)	1.20 (1.09; 1.32)
BW < 3.6 kg **–** *adjusted model 1*	1.27 (1.15; 1.29)	1.20 (1.09; 1.32)
BW < 3.6 kg **–** *adjusted model 2*	1.27 (1.15; 1.40)	1.21 (1.10; 1.33)
Combined model		
BW < 3.6 kg	1.2 (1.16; 1.41)	1.20 (1.09; 1.33)
Childhood OW	1.02 (0.97; 1.08)	0.91 (0.70; 1.18)
Young Adult OW	1.66 (1.41; 1.95)	1.44 (1.20; 1.72)

Hazard Ratios (HRs) were calculated using Cox proportional hazards regression. N = 34,533. The outcome CHD was categorized into early (before 58.4 years of age) and late (after 58.4 years of age). The number of cases among individuals with BW below the median: 861 (early CHD), 837 (late CHD), BW above median: 692 (early CHD), 703 (late CHD). All analyses were adjusted for birth year, country of birth and education level at 45 years of age. Adjusted model 1 was additionally adjusted for childhood overweight, adjusted model 2 for young adult overweight. The combined model included the dichotomized birth weight, childhood and young adult overweight and was adjusted for birth year and country of birth.

CHD, coronary heart disease. BW, birth weight; OW, overweight incl obesity; BMI, body mass index, CI, confidence interval, SD, standard deviation.

DOI [to be added]

Table S8. Adjusted Hazard Ratios for coronary heart disease (CHD) in relation to birth weight and young adult overweight, adjusted for education level

	Early CHD<br>HR (95% CI)	***Late CHD***<br>HR (95% CI)
BW ≥ 3.6 kg/young adult NW	ref	ref
BW < 3.6 kg/young adult NW	1.27 (1.14; 1.40)	1.22 (1.10; 1.35)
BW ≥ 3.6 kg/young adult OW	1.68 (1.36; 2.08)	1.54 (1.22; 1.93)
BW < 3.6 kg/young adult OW	2.18 (1.77; 2.68)	1.58 (1.22; 2.04)

Hazard Ratios (HRs) were calculated using Cox proportional hazards regression. N = 34,533. Number (cases) for BW above median/young adult NW: Early CHD 15,878 (646), Late CHD 13,299 (704); BW below median/young adult NW: Early CHD 16,070 (833), Late CHD 13,331 (834); BW above median/ young adult OW: Early CHD 1,420 (99), Late CHD 1,152 (82); BW below median/young adult OW: Early CHD 1,165 (103), Late CHD 914 (63). All analyses were adjusted for birth year, country of birth and education level.

CHD, coronary heart disease; BW, birth weight; NW, normal weight; OW, overweight incl obesity; CI, confidence interval.

DOI [to be added]

Major Resources Table

In order to allow validation and replication of experiments, all essential research materials listed in the Methods should be included in the Major Resources Table below. Authors are encouraged to use public repositories for protocols, data, code, and other materials and provide persistent identifiers and/or links to repositories when available. Authors may add or delete rows as needed.

Animals (in vivo studies)

Species	Vendor or Source	Background Strain	Sex	Persistent ID / URL
NA	NA	NA	NA	NA

Genetically Modified Animals

	Species	Vendor or Source	Background Strain	Other Information	Persistent ID / URL
**Parent - Male**	NA	NA	NA	NA	NA
**Parent - Female**	NA	NA	NA	NA	NA

Antibodies

Target antigen	Vendor or Source	Catalog #	Working concentration	Lot # (preferred but not required)	Persistent ID / URL
NA	NA	NA	NA	NA	NA

DNA/cDNA Clones

Clone Name	Sequence	Source / Repository	Persistent ID / URL
NA	NA	NA	NA

Cultured Cells

Name	Vendor or Source	Sex (F, M, or unknown)	Persistent ID / URL
NA	NA	NA	NA

Data & Code Availability

Description	Source / Repository	Persistent ID / URL
Anonymized data that are minimally required to reproduce results can be made available from the corresponding author upon reasonable request, and upon approval from the University of Gothenburg according to mandatory national law.	NA	NA

Other

Description	Source / Repository	Persistent ID / URL
NA	NA	NA

DOI [to be added]
